# Docetaxel plus cetuximab biweekly is an active regimen for the first-line treatment of patients with recurrent/metastatic head and neck cancer

**DOI:** 10.1038/srep32946

**Published:** 2016-09-06

**Authors:** Doris Posch, Hannah Fuchs, Gabriela Kornek, Anja Grah, Johannes Pammer, Marie-Bernadette Aretin, Thorsten Fuereder

**Affiliations:** 1 Dept. of Internal Medicine I & CCC, Medical University of Vienna, Währinger Gürtel 18-20, A-1090 Vienna, Austria; 2 Dept. of Pathology, Medical University of Vienna, Währinger Gürtel 18-20, A-1090 Vienna, Austria; 3 Pharmacy Department, General Hospital Vienna, Währinger Gürtel 18-20, A-1090 Vienna, Austria

## Abstract

For patients with recurrent/metastatic (R/M) head and neck squamous cell carcinoma (SCCHN) limited therapeutic options exist. Only a subset of patients is suitable for combination chemotherapy regimens. Biweekly docetaxel plus cetuximab might be an alternative option. Thus, we performed this retrospective analysis in unselected patients in order to investigate the efficacy and safety of this regimen. Thirty-one patients receiving off protocol docetaxel (50 mg/m^2^) plus cetuximab (500 mg/m^2^) biweekly were included. Data collection included baseline demographic, response rate (ORR), disease control rate (DCR), overall survival (OS), progression free survival (PFS) as well as toxicity. OS and PFS were 8.3 months (95% CI 4.8–11.8) and 4.0 months (95% CI 1.0–7.0), respectively. Three (9.7%) patients achieved a complete response and one patient (3.2%) a partial response. The DCR was 41.9% and we observed an ORR of 12.9%. The one-year survival rate was 25.8%. The therapy was well tolerated and the most common grade 3/4 adverse events were neutropenia (19.4%), hypomagnesaemia (12.9%) and acne-like rash (9.7%). Biweekly cetuximab/docetaxel is an effective regimen and well tolerated in R/M SCCHN patients not suitable for platinum doublet treatment. Further evaluation of this regimen in prospective clinical trials is warranted.

Squamous cell carcinoma of the head and neck (SCCHN) accounts for 90% of head and neck cancers and is the tenth most common cancer worldwide^1^. While in the majority of patients alcohol and tobacco consumption is the predominant risk factor for SCCHN development, human papilloma virus infection (HPV) has been identified to contribute to the development of oropharyngeal SCCHN in a subgroup of patients^2^,^3^. A multidisciplinary approach involving radiation oncologists, medical oncologists and head and neck surgeons is necessary for the optimal management of these patients. Nevertheless the locoregional recurrence rate of stage III/IV disease after curative multimodality therapy is about 30–40% in the first 2 years of follow up^4^. Additionally, a constant rate of 2–3% per year of second primaries is observed^5^,^6^. For these patients the treatment options are scarce: In unresectable recurrent or metastatic (R/M) disease palliative poly-chemotherapy is the mainstay of therapy. Despite improvements of treatment regimens and implementation of novel targeted therapies such as the epidermal growth factor receptor (EGFR) antibody cetuximab in the past decade, the median survival time is still 8–10 months^7^. Based on the results of the EXTREME study a combination regimen containing a platinum drug, 5-FU and weekly cetuximab has become standard of care in this setting for patients with excellent performance status^8^. However, a large fraction of patients is not suitable for platinum containing doublet chemotherapy regimens due to an impaired general condition, reduced nutritional status or significant comorbidities. Besides platinum drugs, taxanes such as paclitaxel or docetaxel were demonstrated to be of particular use in this setting. Studies evaluating single agent therapy with docetaxel or paclitaxel reported an objective response rate of 30–40% in chemo-naïve R/M SCCHN patients^9^. Toxicity was shown to be well manageable and mainly restricted to neutropenia or hypersensitivity reactions^9^. From the molecular point of view the combination of taxanes with EGFR targeting agents seems to be beneficial and might exert synergistic activity by various mechanisms such as prevention of taxane induced EGFR phosphorylation or modulation of the EGFR downstream pathways by taxanes^10^,^11^. As for SCCHN one clinical trial showed beneficial effects of a taxane/cetuximab combination regimen after platinum failure^12^. A median overall survival (OS) of 6.7 and a disease control rate (DCR) of 51% was reported^12^. In the first line setting one study is available, which investigated the efficacy of weekly paclitaxel in combination with cetuximab^13^. Since palliation and the maintenance of the quality of life (QOL) is a major goal in this situation, optimizing this treatment schedule administering taxanes plus cetuximab every other week instead of weekly schedules without losing efficacy would be beneficial under patients’ and economic aspects.

Based on this background we performed this retrospective analysis: We investigated the efficacy and safety of upfront docetaxel in combination with cetuximab every other week in patients suffering from R/M SCCHN.

## Patients and Methods

### Data Collection

Patients eligible for this single centre retrospective analysis had histologically or cytologically confirmed R/M SCCHN diagnosed between 1^st^ January 2007 and 30^th^ November 2015 at the Medical University of Vienna. Prior chemotherapy for advanced disease, other than squamous histology and sites other than laryngeal, hypopharynx, oropharynx and oral cavity were exclusion criteria. Previous taxane therapy as part of induction chemotherapy before radiotherapy was allowed.

Demographic and clinical data including patients’ age, ECOG performance status, clinical stage, medical history, tumor response, chemotherapy cycles administered, survival data and toxicity data were collected retrospectively from patients’ notes and prescription charts. The study was performed in accordance with the Declaration of Helsinki and good clinical practice guidelines and was approved by the ethics committee of the Medical University of Vienna (#1643/2016).

### Treatment protocol

Chemotherapy consisted of biweekly docetaxel 50 mg/m2 diluted in 250 ml saline administered as a 90 min intravenous infusion plus cetuximab 500 mg/m2 administered as a 120 min intravenous infusion on day one. Chemotherapeutic/cetuximab treatment courses were repeated every 2 weeks until disease progression, unacceptable toxicity or patient’s request for treatment discontinuation. Ondansetron, dexamethasone and diphenhydramine were routinely given as premedication.

Radiographic imaging employing computed tomography or magnetic resonance imaging was performed at baseline and at 12-weeks intervals until disease progression. Treatment response was evaluated according to RECIST 1.1 criteria by an independent radiologist. Adverse events were graded according to the National Cancer Institute Common Terminology Criteria for Adverse Events (version 4.0).

### Statistical Analysis

Statistical analysis was performed employing SPSS 23 software package (SPSS Inc., Chicago, IL, USA). Continuous variables were shown using descriptive statistics. Categorical variables were summarized using percentages and counts. For survival analysis, including PFS and OS, the Kaplan-Meier method was used for univariate analysis. The data for patients who were alive were censored at the time of last confirmed contact. For response rates two-sided confidence intervals (CI) according to Clopper-Pearson were calculated.

## Results

### Patients and treatment

A total of 31 patients with recurrent/metastatic squamous cell carcinoma of the head and neck were included in this analysis. All patients received front-line chemotherapy with off protocol docetaxel (50 mg/m2) plus cetuximab (500 mg/m2) biweekly until progression or intolerable toxicity. Demographic and clinical baseline characteristics are listed in Table 1. The patient population included 25 (78.1%) male and 6 (21.9%) female patients. The majority of the patients were elderly with a median age of 59 years (range 44–78 years) and a performance status of ECOG 0-1 in 93.5%. The most common primary tumor sites were the oral cavity (35.5%) and the oropharynx (29.0%), followed by the hypopharynx (25.8%) and larynx (9.7%). Three (33.3%) patients with oropharyngeal carcinoma were p16 positive.

While 17 (54.8%) patients suffered from loco-regional recurrence alone, 8 (25.8%) patients were diagnosed with metastasis only (primarily pulmonary metastases). A minority of patients (19.4%) suffered from both loco-regional recurrence and metastatic disease. The majority of patients (87%), was previously treated with surgery, radiation, concomitant chemoradiation or cetuximab based bioradiation.

Alcohol abuse was reported by 17 patients (54.8%) and 26 patients (83.9%) had a history of nicotine abuse (i.e. over 10 pack years). Half of the patients (48.4%) had a body mass index (BMI) of <20 and 51.6% suffered from additional comorbidities.

The median number of chemotherapy cycles with docetaxel plus cetuximab was three (range 1–9). The major reason for chemotherapy cessation was disease progression (93.5%) followed by intolerable toxicity (6.5%).

### Tumor response and survival

Twelve weeks after treatment initiation objective response was assessed by CT scan or MRI according to RECIST 1.1 criteria. We observed three (9.7%) CR, one (3.2%) partial response and 9 (28.1%) stable diseases (Table 2). Thus, the objective response rate was 12.9%, whereas in 13 (41.9%) patients abrogation of progression was achieved.

**Figure 1:**
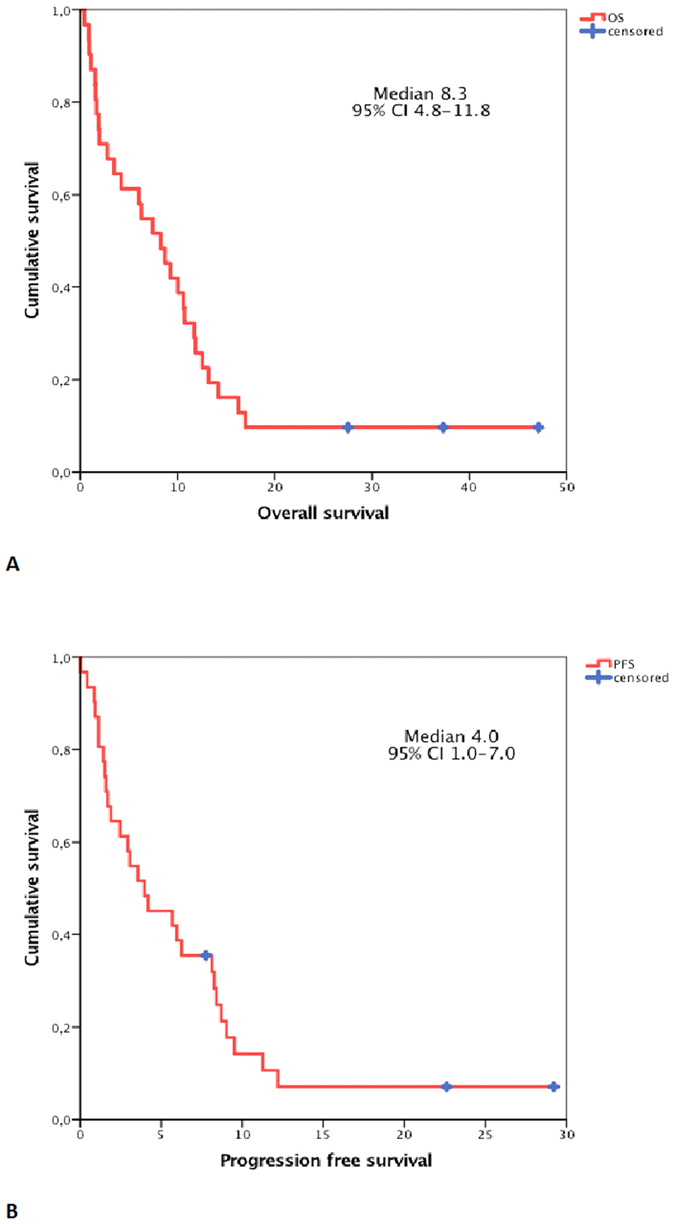
Kaplan-Meier curves depicting overall survival (**A**) and progression-free (**B**) survival.

The median overall survival and progression-free survival were 8.3 months (95% CI 4.8–11.8) and 4 months (95% CI 1.0–7.0), respectively (Fig. 1A,B). 3 patients (9.4%) achieved sustained complete response and are currently alive. The one-year survival rate was 25.8%

### Safety and tolerability

Grade 3–4 adverse events, which were treatment-related, have been observed in 21 patients (67.7%) (Table 3). Neutropenia (19.4%), hypomagnesemia (12.9%) and acne-like rash (9.7%) were the most common ones. Three patients (9.7%) had an allergic reaction to cetuximab. In one case therapy could be continued after treatment with corticosteroids. The other two reactions were life threatening and resulted in discontinuation of cetuximab and patients received docetaxel monotherapy. Pneumonia developed in three (9.7%) patients. Other rare treatment-related grade 3/4 gastrointestinal toxicities and mucositis grade 3/4 were reported in 3.2% and 6.5% of the patients, respectively. One (3.2%) patient suffered from treatment-related severe nausea and one (3.2%) from conjunctivitis grade 3. Grade 1/2 adverse events included onycholysis, hypomagnesemia and diarrhea (data not shown). No treatment-related deaths were registered. Overall docetaxel plus cetuximab was well tolerated and no new safety issues arose.

## Discussion

Advanced R/M head and neck cancer remains a major unresolved health problem. Although intensive efforts are made to improve the overall survival in this patient population, the clinical outcome is still poor. In this retrospective study, we demonstrate that the combination of docetaxel with cetuximab every other week is a feasible, safe and effective regimen in unselected patients suffering from R/M SCCHN.

While the EXTREME study protocol employing a combination of cisplatin/5-FU and cetuximab has become gold standard for the first line treatment of R/M SCCHN patients, only fit patients can tolerate this aggressive regimen^8^. Thus, alternative treatment protocols substituting 5-FU by docetaxel are currently tested in clinical trials. Very recently, a phase II study reported that cisplatin/docetaxel every three weeks plus cetuximab weekly results in an OS of 14 months (95% CI 11.3–17.3) and an ORR of 44%^14^. However, two infectious events leading to death were observed^14^. Additionally, it is well known that 35–65% of head and neck cancer patients are malnourished (i.e. BMI <20), which effects treatment outcome and survival and was also the case in our population (48.4%)^15^,^16^. Administration of combination chemotherapy regimens such as the EXTREME regimen might aggravate this issue. Thus, for a considerable subset of patients single agent chemotherapy plus cetuximab seems to be more appropriate. When cetuximab was introduced, a large phase III trial testing the efficacy and safety of single agent cisplatin plus cetuximab was conducted in R/M SCCHN patients. This study demonstrated that the addition of cetuximab weekly to cisplatin every 4 weeks was superior to cisplatin monotherapy with respect to ORR (26% vs 10%) but exerted in similar OS (9.2 vs. 8.0 months) and PFS (4.2 vs. 2.7 months)^17^. To the best of our knowledge, only one trial is available evaluating the efficacy and safety of a taxane (i.e. weekly paclitaxel) plus cetuximab in this patient population in the first line setting^13^. This study showed a median OS of 8.1 months (95% CI 6.6–9.6 months) and a median PFS of 4.2 months (95% CI 2.9–5.5 months)^13^. Although we are aware that inter trial comparisons have to be interpreted with caution, our findings are in line with the studies mentioned above and the median OS and PFS is comparable. Patients receiving off protocol docetaxel plus cetuximab every other week had a median OS of 8.3 months (95% CI 4.8–11.8) and a median PFS of 4 months (95% CI 1.0–7.0).

Apart from that, tumor response rate is a pivotal issue in the palliative setting. Especially, in SCHNN patients tumor shrinkage often results in symptom relief and improvement in quality of life. Interestingly, the ORR of 12.5% and DCR of 40.6% we observed in our analysis was inferior compared to the paclitaxel/cetuximab trial with an ORR of 54% and a DCR of 80%^13^. However, there is a large variability in the literature with respect to ORR in taxane based trials: While taxane monotherapy (without cetuximab) trials demonstrated an ORR between 27% and 47%, the above mentioned cisplatin/docetaxel plus cetuximab study showed an ORR of 44.4%^9^,^14^,^18^,^19^. So what is the reason for this discrepancy and the low ORR in our analysis? Although- due to the retrospective nature of this analysis and the low patient number- no definitive conclusions can be drawn, it is tempting to speculate that the high fraction of patients (61.3%), who had already received chemo-radiotherapy previously in our study, might have contributed to this inferior ORR. In the paclitaxel/cetuximab trial, the majority of the patients were chemo-naive (59%) and had not already received chemotherapy in advance. The authors found a correlation between tumor response and previous exposure to chemotherapy, which was not translated into a better OS or PFS^13^. This finding is consistent with the results of our study.

With respect to tolerability, no new safety issues arose. This regimen was well tolerated and adverse events were manageable. We observed a similar grade 3/4 adverse event rate (68%) as compared to the paclitaxel/cetuximab study (65%)^13^. The infusion related events (3 vs 2) was almost identical as well^13^.

Apart from efficacy and safety issues, QOL aspects and economic considerations should be taken into account in the palliative setting. It has been proven previously in colorectal cancer and head and neck cancer patients that weekly cetuximab and biweekly cetuximab is equally effective with respect to target regulation, pharmacokinetics and pharmacodynamic parameters^20^,^21^. Additionally, it has been shown in prostate cancer patients that docetaxel biweekly is as effective as docetaxel triweekly, but much better tolerated^22^. Thus, a simplified dosing regimen would be of further value for these patient population improving compliance and eventually QOL. Apart from that, biweekly docetaxel plus cetuximab could help to lower health care costs by reducing the number of hospital visits and the need for health care staff.

Taken together, cetuximab plus docetaxel is a safe and effective regiment as first line treatment in R/M SCCHN patients not suitable for platinum based doublet regimens. A prospective clinical trial is warranted to confirm our data.

## Additional Information

**How to cite this article**: Posch, D. *et al*. Docetaxel plus cetuximab biweekly is an active regimen for the first-line treatment of patients with recurrent/metastatic head and neck cancer. *Sci. Rep.* **6**, 32946; doi: 10.1038/srep32946 (2016).

## Figures and Tables

**Table 1:** Patient and disease characteristics at baseline (please note that comorbidities add up to more than 31 patients, since patients suffered from more than one disease).

Characteristics	Number of Patients (%)
Sex	
Male	25 (80.6%)
Female	6 (19.4%)
ECOG Status	
0–1	29 (93.5%)
2	2 (6.5%)
Median age (range), years	59 (44–78)
Primary tumor site	
Oral cavity	11 (35.5%)
Larynx	3 (9.7%)
Hypopharynx	8 (25.8%)
Oropharynx	9 (29.0%)
p16 status (oropharyngeal carcinoma)	
Positive	3 (33.3%)
Negative	5 (55.6%)
Not evaluable	1 (11.1%)
Alcohol abuse	
No	10 (37.0%
Yes	17 (63.0%)
Not evaluable	4
Nicotine abuse	
No	2 (7.1%)
Yes	26 (92.9%)
Not evaluable	3
Cycles (range)	3 (1–9)
Median duration of treatment	2.1 months
Previous treatment	
Surgery alone	1 (3.2%)
Surgery plus radiotherapy	2 (6.5%)
Surgery plus concomitant chemoradiotherapy	5 (16.1%)
Primary radiotherapy	2 (6.5%)
Primary concomitant chemoradiotherapy	14 (45.2%)
Primary radioimmunotherapy	3 (9.6%)
Radioimmunotherapy	4 (12.9%)
Extent of disease	
Locoregional recurrence alone	17 (54.8%)
Metastatic disease alone	8 (25.8%)
Locoregional recurrence plus metastatic disease	6 (19.4%)
Nutritional status	
Patients at risk of severe weight loss (BMI <20)	15 (48.4%)
Severe weight loss after radiotherapy ( >5% body weight loss in 6 months)	1 (3.2%)
Comorbidities	16 (51.6%)
Myocardial infarction	2
Peripheral vascular disease	5
Chronic obstructive pulmonary disease	6
Diabetes mellitus	4
Polycystic kidney disease	1
Chronic kidney disease or	4
Renal failure during chemoradiation	
Chronic liver disease	4
Stroke	1
Perforation of the stomach	2
Atrial fibrillation	2
Second malignancy treated with curative intent	3

**Table 2:** Summary of treatment results.

Best Response	Number of Patients (n = 31)	Percentage
CR	3	9.7%
95% CI		2.0 to 25.8%
PR	1	3.2%
95% CI		0.1% to 16.7%
Stable Disease	9	29.0%
95% CI		14.2% to 48.0%
Progressive Disease	18	58.1%
95% CI		39.1% to 75.5%
Overall Response Rate	4	12.9%
95% CI		3.6% to 29.8%
Disease Control Rate	13	41.9%
(CR + PR + SD)		
95% CI		24.6% to 60.9%

**Table 3:** Grade 3 to 4 treatment-related adverse events.

Adverse event	Number of patients (%)
All	21 (67.7%)
Neutropenia	6 (19,4%)
Hypomagnesemia	4 (12,9%)
Acne-like rash	3 (9.7%)
Allergic reaction	3 (9.7%)
Pneumonia	3 (9.7%)
Mucositis	2 (6.5%)
Conjunctivits	1 (3.2%)
Nausea	1 (3.2%)
Diarrhea	1 (3.2%)
